# Sympathetic regulation of the host immune response to bacterial sepsis

**DOI:** 10.1042/CS20256909

**Published:** 2025-10-21

**Authors:** Huynh Nguyen, Cameron R. Bastow, Shu Wen Wen, Connie H. Y. Wong

**Affiliations:** 1 Centre for Inflammatory Diseases, Department of Medicine, School of Clinical Sciences at Monash Health, Monash Medical Centre, Monash University, Clayton, VIC, 3168, Australia

**Keywords:** bacterial infections, barrier defence, immune system, sepsis, sympathetic nervous system

## Abstract

Sepsis is a life-threatening condition that occurs when infection drives an overwhelming immune response that damages tissues and results in multi-organ dysfunction. Current treatment of sepsis focuses on eliminating the infectious pathogen and supporting the cardiovascular system. However, effective therapeutics for mitigating the dysregulated immune response in sepsis are still lacking. To this end, many sepsis survivors end up with immunoparalysis and an increased risk of recurring infections. Despite the growing body of research revealing the close interplay between the nervous and immune systems, modulating the neuroimmune pathways remains an unexplored route of treatment. The sympathetic arm of the autonomic nervous system, particularly β-adrenergic receptor signalling, is integral in limiting the inflammatory response during bacterial infections. However, our current understanding of the neuroimmune interactions and their impact on sepsis pathophysiology remains limited. In this review, we outline current insights into the neuroimmune response in sepsis, with a particular focus on the role of the sympathetic nervous system in modulating immune responses against bacterial infections. Elucidating the neural signalling pathways that regulate the immune response and recovery in sepsis will reveal new therapeutic targets to reduce disease burden and improve patient outcomes.

## Introduction

Sepsis is a life-threatening condition caused by the body’s dysregulated response to infection that leads to hypotension and multi-organ failure [1]. The most common causes of sepsis are diarrhoeal disease and lower respiratory infections, both of which are primarily instigated by bacteria [2,3]. Particularly, monomicrobial infections by pathogenic strains of *Escherichia coli* (*E. coli*), *Staphylococcus aureus* (*S. aureus*), *Klebsiella pneumoniae* (*K. pneumoniae*) and *Streptococcus pneumoniae* (*S. pneumoniae*) are most commonly observed in sepsis patients if the causative microbe can be identified [4–6]. To a lesser extent, sepsis can also be triggered by viruses, fungi and parasites [3]. The most recent estimate of the global burden of sepsis predicts 48.9 million cases of sepsis worldwide in 2017, with 11 million sepsis-related deaths. Thus, sepsis accounts for one-fifth of all deaths globally, of which this is likely an underestimation given limitations in data sourced from death certificates [1,3]. Although advances in infection detection [7] and clinical care [8] have led to a decline in sepsis-related mortality, a large proportion of survivors continue to suffer long-term complications known as post-sepsis syndrome [9]. Common complications include sustained immune impairment and physical disabilities, as well as cognitive and mental health issues, which are often an overlooked burden in sepsis care [10,11].

A direct consequence of prolonged immune suppression following sepsis is the increased risk of recurrent infections. One study reported that 63% of sepsis survivors who achieved 30-day survival experienced recurrent infections within a year, which is significantly higher compared with non-septic survivors of other illnesses [12]. Furthermore, these recurrent infections were associated with an increased mortality of 52.7% compared with controls [12]. Similarly, other studies have also observed significantly increased risks of secondary infections and consequently increased risk of death in sepsis patients [13,14]. These observations highlight the need for a deeper understanding of sepsis immunopathology to aid the discovery of novel therapeutic targets to reduce late-stage immune impairment and improve patient outcomes.

Recent research has expanded our understanding of the immune system’s significant interplay with the nervous system in orchestrating defence against bacterial infection. These neuroimmune interactions are crucial to effectively clear pathogens, control inflammation and minimise tissue damage. A key example is barrier immunity, where structural (epithelium) and chemical (mucus) barriers within the skin, lungs and/or gut provide continuous defence against foreign antigens. When pathogens invade these sites, the nervous system triggers downstream signalling pathways to tailor appropriate immune responses, as this review will discuss. Additionally, this review will summarise current findings on the neuroimmune interactions in bacterial infections and sepsis, to identify potential therapeutic targets.

## Sepsis immunopathology

Clinically, organ dysfunction in sepsis patients is determined using the Sequential (Sepsis-Related) Organ Failure Assessment (SOFA) score, which assesses the function of multiple bodily systems, including respiratory, cardiovascular, hepatic, renal and cognitive systems [1,15]. Organ dysfunction in sepsis is defined as an acute increase in total SOFA score of ≥2 points following infection. In some severe cases, sepsis can progress into septic shock despite interventions, marked by cellular, metabolic and haemodynamic abnormalities, including hypotension and elevated serum lactate levels. These life-threatening complications likely arise from excessive production of pro-inflammatory and vasodilatory mediators by the host and the release of microbial toxins. Together, these factors contribute to increased vasodilation, vascular permeability, poor tissue perfusion and, ultimately, organ failure [16,17]. Notably, these events coincide with profound immune alterations, highlighting the critical need to better understand how immune dysregulation contributes to organ failure and mortality in sepsis. A comprehensive understanding of sepsis pathogenesis is essential for the identification and development of effective therapeutic targets.

In a healthy immune response, infections trigger a rapid and co-ordinated response, initiated by the detection of pathogen-associated molecular patterns (PAMPs) and damage-associated molecular patterns (DAMPs), such as lipopolysaccharide (LPS) and nucleic acids, by pattern recognition receptors (PRRs), such as toll-like receptors (TLRs). PRRs are expressed by innate immune cells [18] and non-immune cells, including epithelial [19] and endothelial cells [20]. Upon the activation of PRRs, this leads to the downstream phosphorylation of proteins and the production of inflammatory mediators [19]. In turn, the downstream expression of these mediators, including co-stimulatory molecules which activate and prime T cells and B cells of the adaptive immune response within hours to days [21,22]. The adaptive immune system subsequently mounts a targeted antigen-specific response using antibodies and cell-mediated defences [23]. Under normal circumstances, once the pathogen is effectively eliminated, both the innate and adaptive immune systems return to homeostasis within days to weeks [23]. In sepsis, however, the immune response is profoundly dysregulated, marked by an imbalanced and sustained inflammatory response [24]. When an infection cannot be contained within local tissues, the infection can enter and disseminate through the bloodstream, causing what is known as bacteraemia [25]. This widespread infection further increases the release of PAMPs and DAMPs, overwhelming immune regulatory mechanisms and driving an uncontrolled host immune response characteristic of sepsis [25].

Inflammatory mediators that are commonly elevated during the acute phase of sepsis include interleukins (IL)-1β, IL-6, IL-8, CC motif chemokine ligand 2 (CCL2) and tumour necrosis factor (TNF)-α. The circulating level of these inflammatory mediators in sepsis patients varies depending on factors such as the sepsis stage [26], the presence/absence of bacteraemia [27] and the bacterial species [28]. Nonetheless, the levels of these circulating mediators can reflect the degree of endothelial injury [29], correlate with sepsis severity [28] and can be used to predict 28-day mortality [26]. In a regulated immune response against bacteria, inflammatory mediators are vital for the production of effector molecules such as reactive oxygen species (ROS) and reactive nitrogen species (RNS) to aid bacterial clearance [30]. However, ROS and RNS also function to induce vasodilation; thus, their overproduction during sepsis may lead to severe hypotension, which is a major pathology in sepsis [31–33]. The resulting loss of vascular integrity and impaired blood flow to major organs in the body contributes to multi-organ failure and septic shock [34].

Following a prolonged, excessive inflammatory response, sepsis patients eventually develop into a state of immunosuppression. In a regulated immune response against bacteria [2], an immunosuppressive negative feedback loop is initiated to restore immunological homeostasis after infection to limit excessive inflammation [35]. In sepsis, however, this negative feedback loop is more profound and sustained, resulting in a state of immunoparalysis and increasing the vulnerability of patients to secondary infections. Several processes have been described to contribute to dysregulated immunosuppression in sepsis, including the increased production of anti-inflammatory cytokines, particularly IL-10 [36], increased lymphocyte apoptosis [37], decreased monocyte sensitivity to cytokine signalling [38] and reduced expression of antigen-presentation receptors [39]. Moreover, sepsis triggers massive neutrophil release from the bone marrow, flooding the bloodstream with immature cells and impairing chemotaxis through C-X-C chemokine receptor type 2 (CXCR2) desensitisation, which undermines targeted migration to infection sites [40]. These neutrophils exhibit reduced antimicrobial functions whilst forming excessive, harmful neutrophil extracellular traps (NETs) [41]. Coupled with delayed apoptosis, the resulting heterogeneity of neutrophil subsets drives inflammatory organ injury, immunosuppression and vulnerability to secondary infections [42].

Additionally, studies have identified biomarkers of immunosuppression in sepsis patients, including, but not limited to, a reduced expression of human leukocyte antigen-DR (HLA-DR) and elevated expression of T cell Ig and mucin domain protein 3 (TIM-3) on monocytes [43,44]. A reduction in HLA-DR would indicate the impairment of monocytes in presenting antigens to T cells [45], whilst an elevation of TIM-3 would indicate T cell exhaustion [46]. Furthermore, studies have shown that low expression of HLA-DR was a stronger predictor of developing secondary infections and lower survival [47–49], and likewise for the increased expression of TIM-3 [50]. Overall, persistent immunosuppression remains a major post-sepsis complication with no effective treatment [9]. When sepsis patients are in a state of immunoparalysis, they are more prone to secondary opportunistic and/or nosocomial infections and are unable to mount effective immune responses to clear the infection. Clinically, secondary infection occurs in 13.5–39% of sepsis patients, which lengthens hospital stays, worsens disease outcomes and increases mortality [13,14,51–53]. Together, sepsis immunopathology is driven by an excessive inflammatory response to infection, but even if patients survive, sustained immunosuppression increases their susceptibility to secondary infection and mortality.

## Animal models of sepsis

Animal models of sepsis have been fundamental in our current understanding of the complex mechanisms and sequence of events that underlie the immunopathology of sepsis. However, with the recently renewed definition of sepsis that describes it as life-threatening organ dysfunction caused by a dysregulated host response to infection and operationalised as an acute increase of ≥2 points in the SOFA score [1], there has been a push towards re-evaluating, updating and standardising current sepsis models to be more reproducible and reflective of clinical sepsis [54]. To best recapitulate clinical sepsis, preclinical models should be initiated by infection with clinically relevant pathogens that drive multi-organ dysfunction [54]. By definition, a model that lacks organ dysfunction is not sepsis. Prior to this definition, animal models of sepsis did not routinely assess for multi-organ dysfunction. Traditionally, common preclinical models used to study the host immune response in sepsis include the administration of inflammatory stimuli (e.g. bacterial toxins), inoculation with pathogenic microbes or breaching the gut barrier to release caecal contents and microbes. The strengths and limitations of each of these approaches are discussed below.

### Bacterial toxin administration

The simplest historical model of sepsis is the administration of PAMPs, particularly LPS [55]. This approach is easily adopted and reproducible, allowing researchers to investigate simplified aspects of sepsis, such as the oxidative stress pathways that drive inflammation and hypotension [33]. However, this approach has recently fallen out of favour. Compared to an infection with a live pathogen, this model fails to replicate the host–pathogen interactions resulting from the dynamic expression of PAMPs, DAMPs and virulence factors produced across the time course of infection [56]. Instead, administration of LPS results in rapid and temporal inflammatory cytokine kinetics, which do not reflect the clinical sepsis time course [57,58]. It has also been extensively demonstrated that the response to LPS differs between species, with mice being up to one million-fold more resistant to LPS than humans [59–61]. Additionally, there are differences in how the innate immune system responds to LPS in humans and mice, as shown through transcriptional analyses where mouse macrophages displayed enhanced activation of negative feedback regulators of LPS-induced TLR signalling pathways to reduce the inflammatory response [62]. Furthermore, unlike humans, mice can produce hemopexin, which is an acute-phase protein found in serum that down-regulates LPS-induced pro-inflammatory cytokines from macrophages [63]. Such high doses of LPS required to cause disease in mice are not physiologically relevant in humans, making clinical translation difficult. Thus, it is recommended that researchers adopt alternate models when studying sepsis.

### Caecal ligation and puncture

The caecal ligation and puncture (CLP) model involves invasive surgery on anaesthetised animals to expose and puncture the caecum, resulting in polymicrobial sepsis driven by commensal gut bacteria. Early CLP studies in mice showed clinical features similar to those in human sepsis, such as hypotension, increased pro-inflammatory mediators, reduced leukocyte numbers and signs of organ dysfunction [64]. For these reasons, CLP has long been considered the ‘gold standard’ of a preclinical mouse model of sepsis. However, this approach has several limitations. First, CLP only represents abdominal sepsis, not respiratory or bloodstream infection, and it also lacks source control in that necrotic tissue is not resected, something that would not occur in the clinic. Moreover, this model is highly variable between research groups due to differences in technique, particularly the caecum ligation distance from the puncture point [65], the number and size of punctures [66], and the extent of bacterial dissemination before an abscess forms at the puncture site [67]. Furthermore, the animal gut microbiome varies greatly based on animal housing conditions, which can impact disease outcomes in the CLP model [68]. As such, recent approaches have focused on minimising this variation by establishing stocks of faecal bacteria for direct injection [69]. However, despite attempts to standardise the method to reduce variability, another major limitation in the model is the lack of identification of commensal microbial species driving disease and how they align with known pathogens underlying clinical sepsis [70]. Indeed, the mouse intestinal microflora does not accurately represent the human intestinal microflora [71]. Lastly, whilst the polymicrobial nature of the CLP model is often touted, the majority of clinical sepsis cases are driven by monomicrobial infections by a restricted number of highly pathogenic bacterial species [72–74]. Hence, CLP models do not incorporate the additional contributions to sepsis pathology driven by the pathogenic bacterial species observed in the clinic. Therefore, whilst the CLP model can produce clinical features of sepsis and is responsible for our current understanding of sepsis pathology, the mechanisms behind the model could differ markedly from those operating in clinical sepsis.

### Live bacterial infection

Using live pathogens to induce infection in animal models offers a more accurate and clinically relevant model of sepsis if appropriate precautions are considered. Such considerations include the species and source of the pathogen, the dose and route of infection, and the duration of the disease [58]. For example, intravenous (i.v.) injections can model catheter-facilitated bloodstream infections; intraperitoneal (i.p.) injections can mimic peritonitis and abdominal sepsis; intravesical inoculation can mimic urinary tract infections; and the administration of respiratory bacteria through intranasal or intratracheal techniques can mimic pneumonia-induced sepsis and secondary airway infections. Combining appropriate live pathogens with relevant inoculation approaches in preclinical sepsis models can accurately replicate the range of infection sources observed in clinical sepsis. Whilst many bacterial strains are accessible for research, clinical isolates from sepsis patients are ideal to recapitulate the expression of relevant virulence factors and antimicrobial resistance [75,76].

Needless to say, there are still caveats to using live pathogens to model sepsis. A single bolus of the pathogen is commonly administered to achieve infection rather than the progressive outgrowth of the pathogen typically observed in human infections. This can be due to the animal species not being a natural host for the pathogenic microbe, or necessary virulence factors being species-specific. For example, certain strains of *S. pneumoniae* express the surface protein choline-binding protein A (CbpA), which binds to human complement factor H (FH) to facilitate evasion of the complement system [77]. However, CbpA does not bind to FH in mice, and thus, *S. pneumoniae* cannot evade the complement system and phagocytosis in mice, reducing its virulence [77]. As such, it is important to identify virulent strains that can be used in the animal model. Accordingly, caution must be taken when extrapolating experimental mouse findings to human bacterial infections.

### General improvements to preclinical models

To improve the current models of sepsis to ensure they are clinically relevant, several suggestions have been made. First, extending the progression of sepsis beyond the acute phases of inflammation to study long-term changes seen in clinical sepsis is important. Extending disease duration can be achieved by incorporating standard clinical management of sepsis and septic shock, such as using antimicrobials, vasopressors and fluid resuscitation [78]. Importantly, new therapeutics should be tested with standard care, as this would be the case upon translation. Whilst prophylactic therapies can provide proof of concept, studies identifying new therapeutics should test their efficacy within clinically available treatment windows, specifically after the onset of sepsis symptoms. As sepsis is defined by the suspicion or presence of infection and organ dysfunction, preclinical models should also examine and report the extent of organ damage and dysfunction. Overall, careful interpretation must always be taken when translating animal models to human disease. Given the limitations in each model, preclinical therapeutics should be tested on a variety of sepsis models in different host species to ensure translatability. Despite the drawbacks of current animal models of sepsis, they have been a valuable research tool to enhance our understanding of the pathophysiology of sepsis. The host–pathogen interactions within complex microenvironments following sepsis, and subsequent effects on immune function, cannot be replaced with non-animal methods. Furthermore, the wider implications of sepsis on biological processes beyond the immune response are emerging as an important aspect of patient recovery. Of particular significance is the bidirectional interplay between the sympathetic arm of the autonomic nervous system and the immune system, which is integral in limiting the excessive inflammatory response after bacterial infections. Disruption of this neuroimmune communication can exacerbate immune dysfunction, leading to prolonged inflammation or immunosuppression. Understanding this interplay is therefore critical in discovering new therapeutics for sepsis patients.

## The nervous system

**Figure 1:**
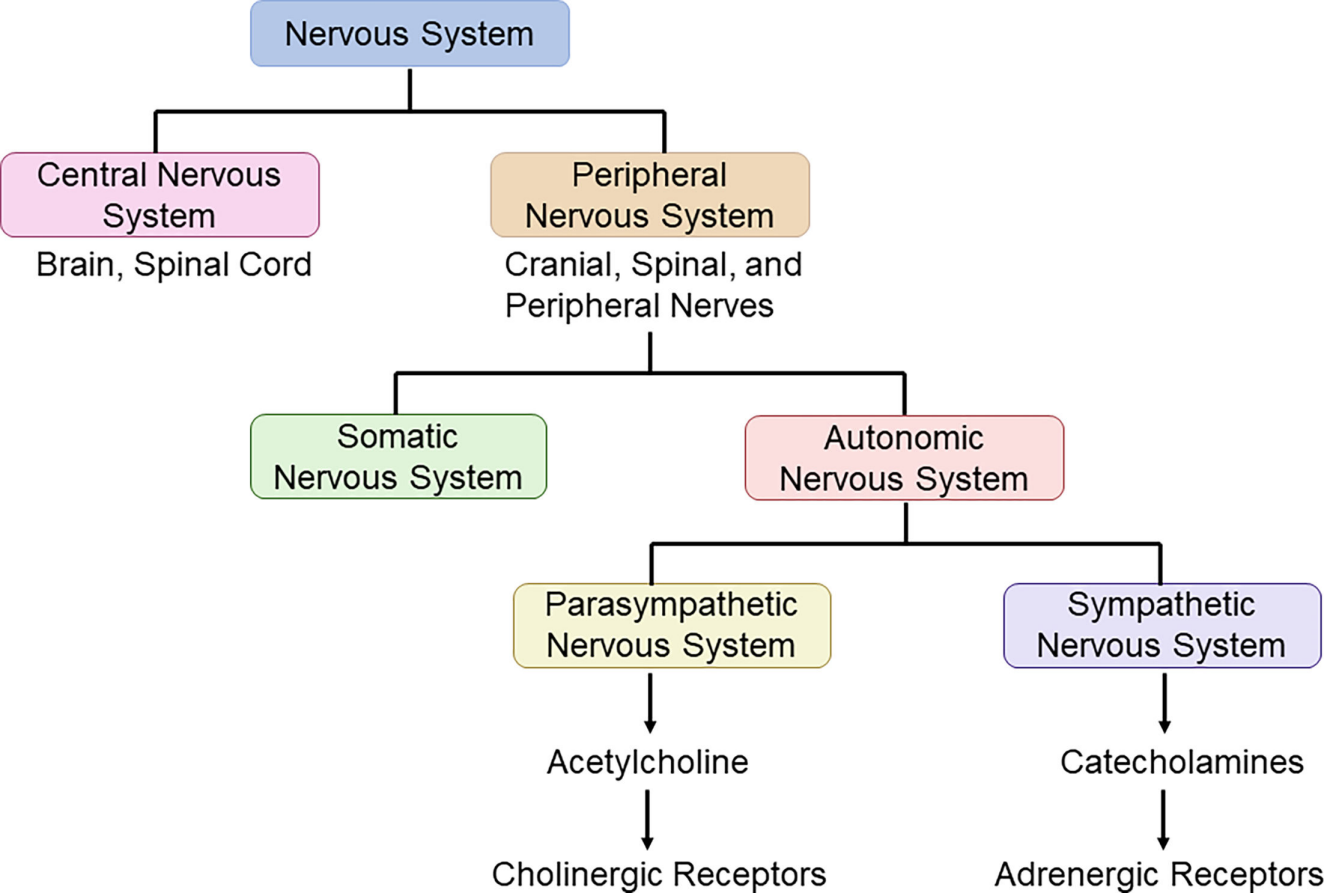
Components of the mammalian nervous system. The nervous system is composed of the central nervous system (CNS), containing the brain and spinal cord; and the peripheral nervous system (PNS), including the cranial, spinal and peripheral nerves. The PNS is further divided into the somatic nervous system, which controls voluntary skeletal movements, and the autonomic nervous system (ANS), which controls involuntary organs. Organs are controlled by the parasympathetic nervous system (PSNS) and sympathetic nervous system (SNS) of the ANS, which release different neurotransmitters (acetylcholine and catecholamines) that are detected by different receptors (cholinergic and adrenergic). Typically, the PSNS controls the resting state of bodily functions, whilst the SNS controls physiological functions of the body to respond to stress.

The nervous system comprises the central nervous system (CNS) and the peripheral nervous system (PNS), which act together to process sensory information and control bodily functions necessary for survival [79]. The CNS consists of the brain and spinal cord, which interact with the nerves of the PNS to facilitate neural control of peripheral organs, glands, muscles and skin [79,80]. The nerve fibres of the PNS are divided into sensory (afferent) and motor (efferent) fibres that conduct signals to or from the CNS, respectively [79]. Motor signals from the CNS signal to skeletal muscles as part of the somatic nervous system, or to the tissues, organs or glands as part of the autonomic nervous system (ANS). The ANS regulates the involuntary control of organs for continuous functioning and is divided into the parasympathetic and sympathetic nervous systems (PSNS and SNS, respectively) (Figure 1). In general, the SNS is activated in moments of stress to increase energy expenditure for survival whilst inhibiting certain physiological functions, such as digestion. Concurrently, the hypothalamic-pituitary-adrenal (HPA) axis is a complex neuroendocrine system that regulates the body’s stress response. When a stressor occurs, the hypothalamus releases corticotropin-releasing hormone that signals the anterior pituitary gland to release adrenocorticotropic hormone, which then stimulates the adrenal cortex to produce glucocorticoid, in the form of cortisol in humans or corticosterone in rodents. In addition to being stimulated by neuropeptides [81,82] or pro-inflammatory cytokines [83], sympathetic signalling in the paraventricular nucleus of the hypothalamus also activates the HPA axis [84]. The co-ordinated network of the PSNS, SNS and HPA axis is central to regulating several bodily functions in response to perceived and systemic stress signals, and returning the body to homeostasis [79,85].

### Mediators of the sympathetic nervous system

**Figure 2:**
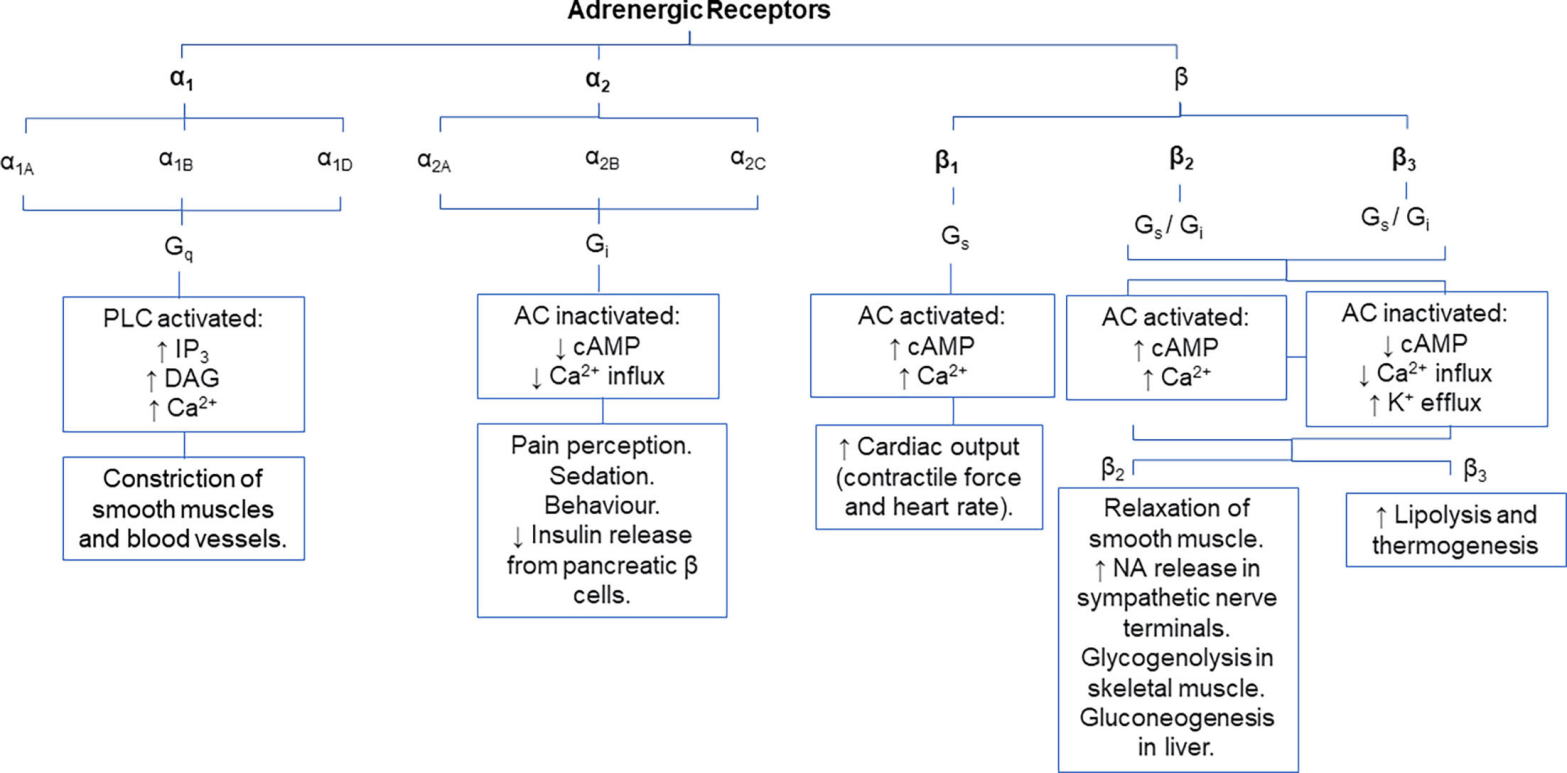
General function of adrenergic receptors. The different subtypes of adrenergic receptors can activate different downstream G-protein signalling pathways, leading to changes in tissue and cell functioning. Abbreviations*:* AC, adenylate cyclase; Ca^2+^, calcium; cAMP, cyclic adenosine monophosphate; CNS, central nervous system; DA, dopamine; DAG, diacylglycerol; IP_3_, inositol trisphosphate; K^+^, potassium; NA, noradrenaline; PLC, phospholipase C.

The main signalling molecules of the SNS are catecholamines, including adrenaline (AD), dopamine (DA) and noradrenaline (NA). Catecholamines bind to specific adrenergic G protein-coupled receptors to activate different signalling pathways that modulate tissue or cellular function. Adrenergic receptors (ARs), classified as α1–2 or β1–3, are expressed differentially across various cell types throughout the body and have distinct affinities for each catecholamine, as previously reviewed by Dunser et al. [86] and summarised in Table 1. Specifically, β1- and β2-AR have similar affinities for AD, but β1-AR has a higher affinity for NA than β2-AR [87]. Additionally, synthetic AR ligands have been developed that demonstrate restricted AR-specific signalling and thus have use as therapeutics to modulate specific AR pathways. However, given the promiscuous binding of endogenous catecholamines to all ARs, the biological response to catecholamines is unlikely in isolation, but determined by the relative binding affinity and the resultant combination of all expressed AR signalling pathways. Indeed, activation of ARs elicits highly context-specific effects based on cell type, tissue and stimulus [88]. Different AR subtypes mediate distinct physiological outcomes driven by their unique downstream signalling pathways, from vasoconstriction and cardiac stimulation to sedation and metabolic regulation, and this has been extensively reviewed in [89] and summarised here in Figure 2. Since the same catecholamine can produce divergent effects depending on the receptor subtype and cellular environment, identifying the specific AR and its expression context is essential for understanding its function [89]. In the context of sepsis, NA is the first-line choice of vasopressor used to treat hypotension in sepsis patients due to its high efficacy in restoring blood pressure with minimal adverse effects on cardiac function [90]. NA binds to β1-AR on myocardial cells to increase contractile force and to α1-AR on vasculature smooth muscle cells to drive vasoconstriction, synergistically increasing blood pressure. Notably, sepsis patients have increased levels of NA in circulation [91]. Thus, treating hypotension with NA can result in the overactivation of the SNS, which has been linked to endothelial damage and increased disease severity [91,92]. Beyond its cardiovascular effects, elevated NA levels in sepsis may also have significant implications for immune regulation.

**Table 1:** Adrenergic receptor ligand specificity discussed in the review

Ligand	α1	α2	α1	α2
Endogenous				
Adrenaline	+	+	+	+
Noradrenaline	+	+	+	+
Dopamine				
<3 μg/kg/minute	-	+	-	-
> 3μg/kg/minute	+	+	+	+
Synthetic				
Agonist				
(R)-(−)-Phenylephrine hydrochloride	+	-	-	-
Dexmedetomidine	-	+	-	-
Salbutamol	-	-	-	+
Isoprenaline	-	-	+	+
Antagonist				
ICI 118,551	-	-	-	+
Alprenolol	-	-	+	-
Atenolol	-	-	+	-
Betaxolol	-	-	+	-
Bisoprolol	-	-	+	-
Carteolol	-	-	+	+
Carvedilol	-	-	+	+
Esmolol	-	-	+	-
Labetalol	+	-	+	+
Landiolol	-	-	+	-
Metoprolol	-	-	+	-
Nadolol	-	-	+	+
Nebivolol	-	-	+	-
Propranolol	-	-	+	+
Sotalol	-	-	+	+
Talinolol	-	-	+	-

‘+’: significant affinity; ‘-’: low/no affinity. Adapted from Table 2 in Dünser and Hasibeder [86].

Sympathetic neurons innervate primary and secondary lymphoid organs such as the spleen, lymph nodes and bone marrow, which enable direct neuroimmune communication [93]. Notably, SNS activation has been demonstrated to impair various immune cell functions, including trafficking and cytokine production [94–97]. While most immune cell types, including macrophages, dendritic cells (DCs), neutrophils, T and B cells, express ARs, the density and subtype distribution can differ significantly across populations [94,98,99]. Additionally, the functional outcome of AR activation is context-dependent, such that different AR subtypes exert opposing effects even within the same immune cell. For example, β2-AR activation typically promotes anti-inflammatory responses in monocytes [96], whereas α1-AR stimulation may enhance pro-inflammatory activity [100]. This receptor subtype-specific signalling allows for multifaceted regulation of immune responses, contributing to the dynamic interplay between the nervous and immune systems during homeostasis and disease states such as bacterial sepsis.

### Preclinical studies of the neuroimmune interactions during bacterial infections and sepsis

Preclinical research has revealed possible biological pathways through which SNS activation may drive dynamic changes to the immune response in sepsis. Activation of the SNS can impact host immunity to infection at various levels, such as the barrier integrity of tissues and organs, the detection of pathogens by the immune system, the expression of inflammatory mediators and the direct activation of immune cells.

#### Neural regulation of barrier defence

The first line of defence against foreign pathogens and bacteria is the physical epithelial barriers that block entry into the body. Such barriers include the skin, respiratory tract and gastrointestinal tract in the body [101]. Epithelial cells form a selective physical barrier that permits the entry of essential products whilst restricting the entry of foreign products and pathogens [102]. Additionally, epithelial cells also contribute to pathogen defence both independently and through interactions with the immune system. Such defence mechanisms include the production of mucus [103] and antimicrobial peptides [104], mucociliary clearance [105] and the phagocytosis of pathogens and their products by immune cells [106,107]. Notably, these barriers are innervated by sympathetic nerves that closely interact with epithelial cells and resident immune cells to detect and respond to stimuli [101]. For instance, SNS activation appears to be important in maintaining epithelial barrier integrity in the gastrointestinal tract, as previously demonstrated in a chemical sympathectomy mouse model [108]. Here, a loss of sympathetic signalling resulted in increased epithelial gut barrier permeability and a pro-inflammatory environment [108]. Interestingly, the study also found that the lack of sympathetic signalling substantially increased tight junction protein expression, including claudin-2 and -3, and occludin [108]. This controversial finding of increased tight junction protein expression despite increased permeability was interpreted by the researchers as an over-compensatory mechanism in which the assembly of tight junctions may have been impaired by the lack of SNS signalling.

Furthermore, nerves in the gut are not static structures; they can expand and retract axons during inflammation and in response to microbes. Indeed, Matheis et al. (2020) demonstrated that enteric bacterial infections reduce neuron density in the ileum, but this damage was reversible via β2-AR–mediated signalling in muscularis macrophages (MMs) to promote neuroprotection [109]. Subsequently, Ahrends et al. demonstrated that pathogen-induced tolerance involves sympathetic neuron activation and β2-AR signalling in MMs, while helminth-triggered neuroprotection occurs through a distinct mechanism [110]. In contrast, a recent study by Tyus et al. [111] demonstrated SNS signalling in the gut drove a hyperinflammatory response in a mouse model of *Clostridioides difficile* infection, as chemical sympathectomy and pharmacological blockade of α2-AR signalling markedly reduced gut inflammation, and decreased disease severity and mortality [111]. The role of SNS in modulating epithelial barrier integrity in the gastrointestinal tract, and perhaps also the respiratory tract, is emerging as an exciting field of research that will drive new discovery and possible therapeutic directions.

During sepsis, the intestinal microbiota is altered such that there is a loss of beneficial microbiota and an overgrowth of potential opportunistic pathogens [112], which can produce factors to increase intestinal permeability and facilitate bacterial translocation [113,114]. Whether the SNS contributes to the disruption of the gut microbiota and bacterial translocation in sepsis is still not well understood. One study investigated the effect of the β1-AR antagonist esmolol on sepsis with the preclinical CLP model in rats, revealing that esmolol improved survival, reduced intraperitoneal TNF-α concentrations and *E. coli* translocation to draining lymph nodes, and prevented damage to the gut mucosa [115]. Future studies should determine whether disrupted SNS signalling modulates the barrier integrity of epithelial cells in the gut, lung and skin in a range of sepsis models.

#### Effects of pathogens on neural signalling

There are many different species, strains and serotypes of bacteria, each differing in the expression of PAMPs and virulence factors, giving them distinctive mechanisms to evade the host immune system and cause infection [116]. Thus, identifying the pathological mechanisms of bacteria is important to formulate appropriate therapeutics. To add another layer of complexity, bacteria can interact with neurons directly, whereby in one study, Gram-positive *S. aureus* infection stimulated sensory neurons to initiate pain sensations and suppress TNF-α release from macrophages [117]. This demonstrates how pathogenic bacterial strains have evolved the ability to exploit neuroimmune regulation pathways, revealing how bacteria can disrupt the normal sympathetic control of the immune response during infections.

In light of this, Straub et al. [118] investigated the role of the SNS in mouse models of bacterial peritonitis and bacteraemia with Gram-negative *Pseudomonas aeruginosa* (*P. aeruginosa*) and Gram-positive *S. aureus* through chemical sympathectomy [118]. When these bacteria were individually injected into mice i.p., sympathectomised mice displayed reduced *P. aeruginosa* burden, but increased burden of *S. aureus* in tissues compared with SNS-competent controls [118]. These results suggest that SNS response to peritonitis may be dependent on differences in virulence factors expressed by the different bacteria. Moreover, in *P. aeruginosa* and *S. aureus* bacteraemia models established by i.v. inoculation, SNS ablation did not affect *P. aeruginosa* burden in tissues, but decreased *S. aureus* organ burden [118]. This highlights the importance of the route of infection when determining the contribution of the SNS during bacterial infections. Whilst the mechanism behind this observation remains uncharacterised, it identifies that the role of SNS activation on immune suppression may be tissue-specific and depend on the extent to which the tissue is innervated by the SNS. Notably, experimental studies using bacteria from clinical bacterial isolates raise the caveat that human-adapted strains may not exhibit similar virulence in murine hosts due to species-specific toxins [77,119]. As a consequence, SNS activation, signalling pathways and immune regulation may not be recapitulated in animal models by humane-adapted strains. Moreover, there may be some differences in SNS activity dependent on the species of bacteria and route of infection; however, this requires further investigation.

#### Neural regulation of inflammatory cytokines

In another study, chemically sympathectomised mice i.v. inoculated with Gram-positive *Listeria monocytogenes* (*L. monocytogenes*) had significantly lower bacterial burdens in the liver and spleen five days post-infection, and a 20-fold higher lethal dose than the vehicle-treated mice [120]. The findings reveal that splenocytes in sympathectomised mice produced greater amounts of interferon (IFN)γ and TNF-α, both of which are critical for reducing *L. monocytogenes* burden in the spleen and liver. In a large animal setting, Lankadeva et al. [121] investigated the role of the abdominal SNS in host antibacterial responses to Gram-negative *E. coli* bacteraemia in sheep [121]. After splanchnic denervation via bilateral splanchnic nerve severing, bacteraemia was induced by i.v. bolus infusion of a clinical *E. coli* isolate. Over 48 hours of monitoring, scarce *E. coli* were detected in the blood of splanchnic-denervated sheep compared with the control sheep with substantially greater *E. coli* burden. Interestingly, these splanchnic-denervated sheep exhibited increased plasma TNF-α and IL-1 levels and reduced IL-10 levels within 6 hours post-infection compared with sham-operated sheep, suggesting the sympathetic regulation of cytokine production that promotes a pro-inflammatory phenotype after infection was mediated via the splanchnic nerve. Indeed, studies ablating the splenic nerve in mice, which is downstream in the signalling pathway from splanchnic nerves, have highlighted that NA production in the spleen inhibits TNF-α release from splenic macrophages [122]. Similarly, stimulation of the vagus nerve during endotoxemia via administration of nicotine, a pharmacological agonist of the nicotinic acetylcholine receptor subunit alpha-7, attenuated TNF production by spleen macrophages in the red pulp and the marginal zone [123]. Whilst the abovementioned studies demonstrated similar cytokine profiles when sympathectomy was performed systemically or via local nerve severing, a recent study revealed activation of SNS locally in the colon via optogenetic stimulation versus systemic administration of NA can result in contrasting colitis outcomes [124]. These studies highlight the importance of dampening inflammation through localised and targeted modulation of the SNS, and perhaps, therapeutic interventions for sepsis need to consider systemic effects of NA in limiting inflammation at the expense of poor bacterial defence.

Supporting this, Stolk et al. [96] investigated the effect of ectopic SNS activation in septic mice using the CLP model of sepsis combined with NA infusion [96]. Here, ectopic SNS activation in septic mice impaired the host response to infection and increased bacterial burden in the spleen, liver and blood after 48 hours compared with control CLP mice without SNS manipulation [96]. A similar reduction in plasma pro-inflammatory cytokines and chemokines, including TNF-α, CCL2 and CCL3, was observed in NA-treated mice, and an increase in anti-inflammatory IL-10 was also observed [96]. Taken together, these studies reveal that the activation of the SNS limits inflammatory responses to the detriment of bacterial clearance. This may be an evolutionary adaptation whereby the immune response can respond rapidly to infections, but requires the activation of the SNS to limit the pro-inflammatory response from causing long-term immune-mediated collateral tissue damage.

#### Neural regulation of innate immune cells

After a physical breach of the epithelial barriers, the first responder to infection is the innate immune system. Innate immune cells express various ARs with the capability to respond to SNS signals during an infection [94,98]. For instance, one study investigating murine bone marrow-derived neutrophils identified the gene expression of all AR subtypes, with the highest expression of β2-AR [94]. In another study by Howell et al. [98], a wide range of bovine immune cells were shown to express various levels of ARs [98]. AR signalling mediates different functional changes dependent on the AR subtype expressed and the innate immune cell type, as we will discuss in the following sections and summarised in Table 2.

**Table 2:** Characterised effects of adrenergic manipulation on innate immune cells during inflammatory settings

Immune cell	Host	Receptor	Ligand	Effect	Authors
Monocytes	Human	α1-AR	(R)-(−)-Phenylephrine hydrochloride	After LPS stimulation (*in vitro*): ↑ IL-1β ↑ PKC-dependent p38 MAPK phosphorylation = ↑ pro-inflammatory cytokine production	[100]
Monocytes	Human	β2-AR (specific antagonist reversed effects of ligands)	Noradrenaline	After various TLR ligands and heat-killed bacteria: ↓ TNF-α, IFN-γ-induced protein 10, IL-1β ↑ IL-10	[96]
Bone marrow-derived macrophages	Mouse (C57BL/6J)	β2-AR (specific antagonist reversed effects of ligands)	Noradrenaline	After various TLR ligands stimulation (*in vitro*): ↓ TNF-α, IL-12, IL-6 ↑ IL-10	[97]
Peritoneal macrophages	Mouse	β2-AR (specific antagonist reversed the effects of the ligand)	Adrenaline; noradrenaline	After LPS stimulation (*in vitro*): ↑ M2 ‘anti-inflammatory’ phenotype: ↑ arginase-1, IL-10, GM-CSF iNOS, IL-12p40, TNF, MIP-1α CXCL1, CXCL2 chemokines phagocytic activity	[95]
Dendritic cells	Mouse (C57BL/6J)	α2-AR	Dexmedetomidine	After LPS stimulation (*in vitro*): ↓ antigen processing ↓ MHC-II (I-A^b^) and CD86 ↓ migration	[125]
Dendritic cells	Mouse (C57BL/6J)	β2-AR	Salbutamol	After LPS stimulation (*in vitro*): ↑ IL-10 ↓ IL-12p70	[126]
Dendritic cells	Sprague-Dawley rats	β-AR	Isoprenaline	After LPS stimulation (*in vitro*): ↓ MHC-II and CD86 ↑ antigen uptake ↑ IL-10	[127]
Neutrophils	Human	β2-AR	Adrenaline	After LPS stimulation (*in vitro*): No effect on IL-6, IL-8 and TNF-α Inhibited CD54 after 6 h ↑ CD44 after 12 h	[128]

MAPK, mitogen-activated protein kinase. MHC, major histocompatibility complex. GM-CSF, granulocyte-macrophage colony-stimulating factor. iNOS, inducible nitric oxide synthase. PKC, protein kinase C. MIP, macrophage inflammatory protein. CXCL, C-X-C motif ligand.

##### Monocytes/Macrophages

Monocytes are derived from the bone marrow and circulate in the bloodstream, from which they can enter tissues to differentiate into DCs or macrophages [129]. Macrophages reside within the majority of organs, exhibit high plasticity in response to their microenvironment and perform specialised functions including tissue surveillance, antigen presentation to adaptive immune cells [130] and tissue repair/remodelling [131]. Grailer et al. [95] investigated how catecholamines alter the phenotype of activated murine macrophages [95]. LPS stimulation alone induced higher inducible nitric oxide synthase (iNOS) expression, which is indicative of an M1-like phenotype [95]. Conversely, co-stimulation of LPS-activated peritoneal macrophages with AD and NA promoted an M2-like phenotype, as indicated by increased expression of arginase-1, IL-10 and granulocyte-macrophage colony-stimulating factor (GM-CSF), while reducing pro-inflammatory mediators including IL-12p40, TNF, CCL3 and chemokines C-X-C motif ligand (CXCL)1 and CXCL2 [95]. This effect was mediated through β2-AR, as the β2-AR-specific antagonist ICI 118,551 reversed the effect of AD and NA [95]. This suggests that catecholamine activation through β2-AR can drive anti-inflammatory responses of macrophages. Similarly, Ağaç et al*.* also demonstrated that NA signalling through β2-AR on murine bone marrow-derived macrophages rapidly induced anti-inflammatory IL-10 production [97]. This mechanism translates to human monocytes, as Stolk et al. [96] demonstrated that NA had anti-inflammatory roles in primary human monocyte cultures activated with LPS by reducing TNF-α, CXCL10 and IL-1β production while increasing IL-10 levels [96]. Similar results were observed when human monocytes were stimulated with other PAMPs, including Pam3Cys (TLR1/2 agonist), Poly I:C (TLR3 agonist), flagellin (TLR5 agonist) and R848 (TLR7/8 agonist), and bacteria such as *E. coli*, *S. aureus* and *P. aeruginosa* [96]. Furthermore, pre-incubating primary human monocytes with a range of AR antagonists confirmed that anti-inflammatory effects were mediated through β2-AR [96].

While these studies have shown that β2-AR activation in monocytes elicits an anti-inflammatory phenotype, this was not seen with α1-AR stimulation. Grisanti et al. [100] found that α1-AR stimulation on primary human monocytes and monocyte-derived macrophages significantly increased IL-1β production in both cell types following LPS stimulation compared with cultures treated with either the α1-AR agonist or LPS only [100]. This increase in IL-1β production was found to occur through the activation of p38 mitogen-activated protein kinase (MAPK) [100]. As the activation of α- and β-ARs can lead to different signalling pathways in monocytes/macrophages, it would be worth investigating how other signalling molecules in the distinctive pathways of each receptor could contribute to either the pro- or anti-inflammatory outcome seen.

##### Dendritic cells

DCs are antigen-presenting cells critical for initiating adaptive immune responses [132]. Typically, they capture and process antigen for surface presentation on major histocompatibility complex (MHC) molecules, and together with the expression of costimulatory molecules, cluster of differentiation (CD)80 and CD86, activate antigen-specific naïve T cells. DCs are also classified according to their haematopoietic origin, anatomical location and surface markers expressed, as previously reviewed [133]. Stimulation of α- and β-ARs on LPS-activated DCs largely promotes an anti-inflammatory response. For example, in studies investigating rodent bone-marrow-derived DCs, β2-AR stimulation with either salbutamol or isoprenaline significantly increased IL-10 production after LPS exposure compared with LPS treatment alone [126,127]. Furthermore, α2-AR stimulation with dexmedetomidine or β2-AR stimulation with isoprenaline significantly reduced the expression of MHC-II and CD86, suggesting an impaired capacity of DC antigen presentation to T cells [126,127]. Thus, contrary to monocytes and macrophages, where anti-inflammatory effects are predominantly driven by β2-AR signalling, both α- and β-AR activation induce an anti-inflammatory response in DCs. Given that these studies only investigated bone marrow-derived DCs in culture, further studies are needed to verify the impact of SNS activation on the inflammatory functions of DCs *in vivo*. In particular, it would be important to investigate the effect of α- and β-AR signalling on the function of different subtypes of lymphoid-resident DCs, tissue-resident DCs that migrate to lymphoid organs upon activation and monocyte-derived DCs that are recruited to sites of infection.

##### Neutrophils

Neutrophils are the most abundant leukocytes in the human body and play critical roles in antimicrobial defence. They primarily achieve this by phagocytosing pathogens, releasing inflammatory and antimicrobial mediators through degranulation, and forming NETs to capture and eliminate pathogens [134]. Seldom have studies investigated the direct role of AR signalling on neutrophils in inflammatory conditions. Instead, most research has focused on the effects of AR signalling on neutrophils through *in vitro* settings. For example, Nicholls et al. [94] demonstrated that treatment of murine bone marrow-derived neutrophils with NA *in vitro* impaired neutrophil chemotaxis and down-regulated the expression of various pro-inflammatory genes, including *Icam1*, *Myd88*, *Mmp9* and *Tnf* [94]*.* Wahle et al. [128] investigated the effect of varying doses of AD on β2-AR signalling in human neutrophils activated by LPS [128]. Here, they found no effect on pro-inflammatory cytokine expression and only minimal effects on the expression of adhesion molecules, suggesting that AD may be more important for modulating neutrophil chemotaxis instead of cytokine expression [128]. The activation of different ARs with different ligands and their roles in modulating the response of neutrophils to different inflammatory stimuli requires further investigation.

### Neural regulation of adaptive immune cells

Extensive research has identified functional impairment of adaptive immune cells among sepsis patients. Robust clinical evidence indicates that patients with sepsis experience significantly reduced circulating B and T cells [37,135–138]. The remaining lymphocyte populations display an exhausted phenotype consistently associated with immunosuppression, increased mortality and worsened clinical outcomes [37,135–138]. Both B and T cells in humans and mice express ARs, primarily the β2-AR subtype [98,99,139]. Preclinical studies have suggested that SNS signalling may suppress the antimicrobial and inflammatory responses of lymphocytes [140], induce T cell exhaustion [141] and promote lymphocyte apoptosis [142]. Despite this, very limited studies have investigated the effect of SNS signalling on lymphocytes in the context of bacterial sepsis. Nevertheless, a study by Ben-Shalom et al. [143] showed that β2-AR signalling on primary cultures of human B cells promoted cell motility and binding affinity of immunoglobulin (Ig) G to viral antigens, but reduced the expression of surface IgG and clonal expansion [143]. Alarmingly, non-surviving sepsis patients often exhibited low B cell counts [144], and the low serum Ig levels were associated with high mortality [145,146]. Indeed, reduced memory B cells in sepsis patients contribute to immunosuppression [147], and impaired CD4^+^ T cell memory responses have been observed in preclinical sepsis models [148]. Interestingly, a recent study demonstrated that SNS activation is crucial for generating memory CD4^+^ T cell responses to *S. aureus* [149]. Here, chemically sympathectomised mice exhibited significantly reduced CD4^+^ T cell recruitment to the infection site upon secondary exposure to *S. aureus* compared to SNS-sufficient controls [149]. Additionally, a recent study found that exhausted CD8^+^ T cells from chronically infected mice cluster near SNS neurons in a β1-AR-dependent manner [141]. Forced overexpression of *Adrb1* in CD8^+^ T cells via a retroviral vector markedly reduced pro-inflammatory cytokine production and proliferation upon catecholamine exposure *in vitro* [141]. Conversely, mice deficient in β1-AR were protected from terminal T cell exhaustion during chronic viral infection [141]. These findings highlight how SNS signalling can modulate T cell fate during chronic infection, raising important questions about its broader impact on lymphocyte function and host defence. Despite this, the mechanisms of adaptive immune exhaustion in bacterial sepsis are incompletely understood, though similar SNS mechanisms that drive T cell exhaustion in chronic viral infection may also be present in bacterial sepsis, but this needs to be explored.

Resolution of inflammation is elicited by multiple signalling and cellular negative feedback loops to restore balance and promote tissue repair [35]. Regulatory T cells (Tregs) play a key role in the context of *Listeria* infection, peaking at 24 hours after infection and again at seven days, thereby reducing inflammation and protecting tissues from collateral damage [150]. In addition to modulating adaptive immune responses, Tregs also influence innate immune cells, such as macrophages and neutrophils, via promoting anti-inflammatory phenotypes [151,152]. Indeed, clinical studies have identified that septic shock patients have increased Treg proportions, and this elevation is associated with sepsis-induced immunosuppression [153,154]. Experimentally, Tregs have been shown to express β2-AR, and stimulation of β2-AR on Tregs enhanced their suppressive effects as indicated by increased expression of inhibitory receptor, cytotoxic T-lymphocyte associated protein 4 (CTLA-4) and higher conversion of naïve T cells into induced Tregs [155], again highlighting the suppressive role SNS activation has to limit inflammation.

Taken together, there is a growing body of work to indicate that the activation of SNS could contribute to impaired adaptive immune activation, memory responses, promote immunosuppression and exhaustion, and this is summarised in Table 3. The effect of SNS modulation of the adaptive immune response would be impaired clearance of pathogens and emergence of opportunistic infections or latent virus reactivation due to impaired memory responses. However, the precise mechanistic insights of how SNS signalling in bacterial sepsis regulates the crosstalk between innate and adaptive immune response for effective antibacterial defence and immune memory require further exploration.

**Table 3:** Characterised effects of adrenergic manipulation on adaptive immune cells

Immune cell	Host	Receptor	Ligand	Effect	Authors
B cells	Mouse; human	β2-AR (specific antagonist reversed the effects of the ligand)	Noradrenaline; metaproterenol	After procedural stress induction (*in vivo*) for mice, e*x vivo* culture of B cells from SARS-CoV-2 donors: ↑ IgG binding affinity to antigen ↓ surface IgG expression ↓ clonal expansion ↑ cell motility	[143]
CD4^+^ T cells	Mouse	Unspecified AR subtype	Noradrenaline (endogenous)	Chemical sympathectomy followed by *S. aureus* infection (*in vivo*): ↓ germinal centre formation ↓ number of CD4 + T cells at the site of infection	[149 *]*
CD8^+^ T cell memory	Mouse	β1-AR (specific antagonist and genetic deficiency reversed the effects of the ligand)	Noradrenaline	Overexpression of β1-AR followed by catecholamine exposure (*in vitro*): ↓ pro-inflammatory cytokine expression ↓ proliferation ↑ exhaustion markers	[141]
Regulatory T cells (Treg, CD4^+^Foxp3^+^)	Mouse	β2-AR (specific antagonist reversed the effects of the ligand)	Noradrenaline	Pre-treatment with noradrenaline, washed and co-cultured with naïve T cells (*in vitro*); ↓ naïve T cell proliferation ↑ conversion of naïve T cells into induced Tregs ↑ CTLA-4	[155]

SARS-CoV-2, severe acute respiratory syndrome coronavirus 2. CTLA-4, cytotoxic T-lymphocyte associated protein 4. Ig, immunoglobulin.

### Remaining gaps in knowledge

Overall, the majority of research into the impact of AR signalling on immune cells during inflammation to bacterial components has shown that β2-AR signalling results in an anti-inflammatory response. Alternatively, α-AR signalling results in different inflammatory responses depending on the immune cell type, where α-AR activation promotes pro-inflammatory responses by monocytes and macrophages, and anti-inflammatory responses by DCs. Whilst understanding the outcome of receptor-specific AR signalling on immune cell function is beneficial for the development of therapeutics to modulate SNS signalling in sepsis, it remains unlikely that a single AR pathway is activated in immune cells *in vivo,* given their expression of multiple AR subtypes and the promiscuous binding of endogenous catecholamines to all AR subtypes. Additionally, whilst *in vitro* and *ex vivo* systems have been instrumental in elucidating cellular and molecular adrenergic signalling pathways in modulating the immune response, the impact of SNS activation on innate immune cell function following the resolution of infection *in vivo*, leading to immunoparalysis, remains unclear. Furthermore, most studies have investigated the use of externally introduced AR stimulators. There is a need to examine the effect of endogenous catecholamine expression on innate immune cells during infection, and whether this is perturbed during sepsis. To understand the specific impact of AR stimulation on immune cells, inducible, conditional AR-knockout models need to be generated, such as those utilising the tamoxifen-inducible Cre-LoxP system [156]. This system enables temporal and spatial gene deletion, such that ARs can be deleted at specific time points in an animal model, and targeted to specific cell subsets by tissue/cell-specific promoters. Currently, tamoxifen-inducible Cre-LoxP models for deleting AR in mice are not commonly used in studies of infection and inflammation. Once it is known how the SNS is functioning on innate immune cells in preclinical models, therapeutics targeting the neuroimmune response can be developed for sepsis.

## Therapeutics targeting SNS signalling in clinical sepsis

Emerging evidence suggests that targeting the SNS offers a promising therapeutic strategy to modulate the immune response. For instance, clinical studies have investigated the use of dexmedetomidine (DEX), an α2-AR agonist typically used to relieve sepsis patients of the pain and agitation felt when receiving invasive interventions, such as mechanical ventilators or intravascular catheters [157,158]. A meta-analysis of clinical trials using DEX to treat sepsis patients in intensive care units (ICU) found that its use significantly reduced IL-6, TNF-α and overall mortality [159]. This immunomodulatory effect of DEX was also observed in a CLP mouse model, in which DEX-treated sepsis mice had significantly lower concentrations of IL-6 and IL-1β in the blood and hippocampus [160]. Despite encouraging findings, studies observing benefits of DEX in sepsis did not attribute them to anti-inflammatory effects [161], suggesting that its benefits may extend beyond immune modulation and involve broader effects on SNS signalling on other bodily systems. For instance, the wider benefit of DEX was demonstrated in a preclinical study by Lankadeva et al. [162], whereby DEX administration reduced the dose of NA required to preserve renal function in septic sheep with acute kidney injury [162]. Similarly, a few studies have demonstrated that the use of β2-AR agonist, salbutamol, in CLP and LPS models of sepsis reduced systemic inflammation and organ damage to promote the overall survival of the mice [163,164]. However, more thorough research on the use of β-AR agonists should be performed in animal models of sepsis induced by live bacterial infections, whilst mimicking clinical sepsis care and treatment to determine the therapeutic window during which their use would be most beneficial. The timing of when AR agonists are given to sepsis patients is important, as their potential anti-inflammatory function may not be beneficial for recovering patients with weakened immune systems.

While current research has predominantly focused on attenuating the immune response in early sepsis, more studies are required to explore the impact of therapeutic immunosuppression on the later stages of sepsis, where there is a need to reactivate the immune system during sepsis-induced immunoparalysis. A landmark study by Stolk et al. [96] revealed that one-third of septic shock patients in ICU with pre-existing use of β-AR antagonists (β-blockers), likely for regulating their cardiovascular functioning, exhibited a significantly higher pro-inflammatory TNF-α to anti-inflammatory IL-10 ratio than those not on β-blockers. Having a higher TNF-α to IL-10 ratio is beneficial in sepsis survivors with weakened immune systems, as an earlier clinical study showed that a sustained overproduction of IL-10 is a predictor of sepsis severity and worsened outcomes [165]. These results suggest that in the absence of β-blockers, NA signalling through β-AR promoted an anti-inflammatory response in patients, similar to observations in preclinical experiments as previously discussed. As such, prolonged administration of NA for haemodynamic stabilisation may contribute to the weakening of the immune system in recovering sepsis patients. Research should be directed to the use of AR blockers to suppress SNS activation and whether it can reactivate the immune system in sepsis survivors with immunoparalysis.

Many clinical studies have investigated the use of β-blockers in sepsis patients, particularly on their safety and efficacy on cardiovascular function and 28-day mortality, rather than immune response, as summarised in Table 4. Studies generally observed that β-blocker usage reduced 28-day mortality by up to 31.1%, highlighting the efficacy of β-blockers in treating sepsis patients [166,168–171]. Additionally, a population-based study by Hong et al. [167] also revealed that hypertensive patients who were already receiving non-selective β-blockers 90 to 365 days before the diagnosis of sepsis had a lower risk of sepsis than non-users, whilst sepsis patients treated with non-selective β-blockers within 90 days of being diagnosed had a higher risk of sepsis than non-users [167]. In a study by Cocchi et al. [169], sepsis patients in ICU who were not previously on β-blocker therapy were treated with β1-AR antagonist esmolol [169]. Here, there was no impact on inflammatory cytokine production, mortality, septic-shock-free days or the proportion of patients on renal replacement or mechanical ventilation therapy compared with those on standard sepsis care without esmolol [169]. Why some sepsis patients benefited from β-blocker therapy whilst others did not may be due to differences in when they started receiving β-blocker treatment. Given that β-AR signalling drives anti-inflammatory responses, prophylactic blocking of β-ARs in patients before sepsis diagnosis may enhance the pro-inflammatory response to infection, thus restricting infection severity. Thus, the therapeutic window is likely missed when administering β-blocker therapy to sepsis patients after the causative infection is established. These hypotheses require further investigation.

**Table 4:** Clinical studies investigating the safety and efficacy of β-blockers in sepsis patients

Published	Study site	Intervention	Outcomes	Authors
Sep. 2024	Medical Information Mart for Intensive Care (MIMIC)-IV, Beth Israel Deaconess Medical Center in Boston, Massachusetts, USA, between 2008 and 2019	Selective β1-blockers: esmolol and metoprolol Non-selective α- and β-AR blocker: labetalol Administered within 48 hours of enrolment	-5.6% reduced 28-day mortality. Longer length of stay (LOS) in ICU (+0.88 days) and hospital (+1.07 days) Reduced 28-day cumulative ventilation-free days (-0.37 days) Increased 28-day cumulative vasoactive agent-free days (+0.65 days) Patients with pre-existing cardiovascular disease benefited most from β-blockers	[166]
Apr. 2023	National Health Insurance Research Database (NHIRD) of Taiwan, between 2000 and 2010	Selective β1-blockers: atenolol, bisoprolol, metoprolol, betaxolol, esmolol Non-selective β-blockers: propranolol, carteolol, nadolol, alprenolol, sotalol Administration was either current (treated within 90 days of sepsis diagnosis) or recent (treated 90 to 365 days before sepsis diagnosis)	Current and recent users of selective β-blockers had a lower risk of sepsis than non-users Current users of non-selective β-blockers had a higher risk of sepsis than non-users Recent users of non-selective β-blockers had a lower risk of sepsis than non-users	[167]
Feb. 2023	Tianjin Third Central Hospital, Tianjin, China	Esmolol Administered 24 hours after sepsis diagnosis	-18% reduced 28-day mortality and -14% reduced 90-day mortality. No difference in the duration of mechanical ventilation use No difference in LOS in ICU and hospital	[168]
Apr. 2022	Beth Israel Deaconess Medical Center and Lahey Hospital and Medical Center, Boston, Massachusetts, USA	Esmolol Sepsis patients who had no prior treatment with intravenous β-blockers.	No difference in mortality No difference in septic shock-free days No difference in the proportion of patients on renal replacement therapy or mechanical ventilation Lower levels of C-reactive protein No difference in IL-4, IL-6, IL-10 or TNF-α	[169]
Oct. 2020	54 participating hospitals in Japan	Selective β1-blocker: Landiolol Administered to patients admitted to ICU within 72 hours	-8% reduced 28-day mortality Improved heart rate -16% lower occurrence of new-onset arrhythmia +4.1% occurrence of adverse effects	[170]
Oct. 2015	Greifswald University Hospital, Greifswald, Germany	Selective β1-blockers: atenolol, bisoprolol, metoprolol, nebivolol, talinolol Non-selective β-blockers: carvedilol, propranolol Non-selective β-AR blocker: sotalol Continued, pre-existing, chronic oral β-blocker usage	Discontinuation of β-blocker treatment increased 28- and 90-day mortality ICU LOS was longer for patients on continued use of β-blockers No difference in hospital LOS	[171]

LOS, length of stay. ICU, intesive care unit.

## Future directions

With emerging research, our understanding of sepsis continues to evolve, particularly in elucidating the role of the nervous system in contributing to sepsis immunopathology. Animal models of sepsis and *in vitro* cell studies have provided evidence for neuroimmune regulation of the host defence against bacterial infections. Previous clinical studies have investigated the use of sympathetic modulators in sepsis patients and found significant reductions in mortality; however, the underlying mechanisms behind their benefits remain unclear. This knowledge gap is exemplified by the opposing clinical outcomes observed with the use of β-AR blockers on sepsis patients before or after diagnosis. Thus, future studies should determine the role of the SNS in modulating the host response to sepsis, specifically utilising clinically relevant preclinical models of sepsis conforming to updated definitions of sepsis and suggestions to standardise preclinical approaches. Additionally, experimental approaches should utilise bacterial infection models that span different species, strains and routes of infection to reflect the heterogeneity of clinical sepsis and further elucidate microbe-specific effects on SNS signalling previously identified. We propose the following gaps in knowledge should be addressed: 1) how the SNS modulates our defence barriers during bacterial infections, 2) how endogenous SNS signals modulate the immune response during sepsis, 3) the immune cell-intrinsic functions of SNS signalling during sepsis through the generation of new inducible, conditional AR-deficient genetic animal models, and 4) the therapeutic efficacy of AR agonism/antagonism in clinically achievable therapeutic windows in preclinical models of sepsis with standard-care practices. A greater understanding of these mechanisms will advance the development of novel neuroimmune therapeutics to reduce sepsis disease burden and improve survival and quality of life for patients.

## Abbreviations

AD: adrenaline
ANS: autonomic nervous system
AR: adrenergic receptor
CCL: CC motif chemokine ligand
CD: cluster of differentiation
CLP: caecal ligation and puncture
CNS: central nervous system
CTLA-4: cytotoxic T-lymphocyte associated protein 4
CXCL: C-X-C motif ligand
CXCR: C-X-C chemokine receptors
CbpA: choline-binding protein A
DA: dopamine
DAMP: damage-associated molecular pattern
DC: dendritic cell
DEX: dexmedetomidine
FH: factor H
HLA-DR: human leukocyte antigen-DR
HPA: hypothalamic-pituitary-adrenal
ICU: intensive care unit
IFN: interferon
IL: interleukin
Ig: immunoglobulin
LOS: length of stay
LPS: lipopolysaccharide
MAPK: mitogen-activated protein kinase
MIP: macrophage inflammatory protein
MM: muscularis macrophages
NA: noradrenaline
NET: neutrophil extracellular trap
PAMP: pathogen-associated molecular pattern
PKC: Protein kinase C
PNS: peripheral nervous system
PRR: pattern recognition receptor
PSNS: parasympathetic nervous system
RNS: reactive nitogen species
ROS: reactive oxygen species
SARS-CoV-2: severe acute respiratory syndrome coronavirus 2
SNS: sympathetic nervous system
SOFA: Sequential Organ Failure Assessment
TLR: toll-like receptor
TNF: tumour necrosis factor
Tregs: regulatory T cells
iNOS: inducible nitric oxide synthase
